# Strategies for remote assessment of medical students at University of Minho

**DOI:** 10.1111/medu.14322

**Published:** 2020-08-28

**Authors:** José Miguel Pego, Joana Almeida Palha, Peter Vincent Scoles, Nuno Sousa

## 1 WHAT PROBLEMS WERE ADDRESSED?

The outbreak of the COVID‐19 pandemic was extraordinarily disruptive and presented medical educators with unprecedented challenges. The School of Medicine at University of Minho rapidly moved most curriculum activities to online formats, including our assessment processes. Our experience in remote assessment was very limited when we made the decision, and we knew that there was not enough time for extensive pilot testing. We were confident that we could leverage our overall expertise in assessment design and delivery, and we were able to gather valuable information access to and confidence with web‐based instruments through student surveys.

## 2 WHAT WAS TRIED?

Recognising the various critical dimensions for valid remote assessment, the School of Medicine at University of Minho adopted the following parameters for remote assessment:

*Online examinations will be formative assessments*. These examinations are designed to help students measure individual progress, and allow faculty to monitor the effectives of a remotely delivered curriculum and assessment process. There will be adequate student training for the assessment process and prompt feedback on performance.

*Item characteristics*. Our examinations emphasise integration and problem solving rather than recall. Items are timed to minimise reliance on external sources. Test items are presented in random order to further protect security, and we trust students to act with integrity.

*Fairness*. Fairness is a key aspect in assessment. As we cannot assure uniformity of testing conditions for remote examinations, and we do not know yet the impact of variable testing circumstances on exam performance, we decided to normalise scores to the average score of the past 3 cohorts in the same examination. This is based on the premise that each cohort of students is quite similar in terms of average performance. Student performance will be calculated as a z‐score around average performance of the remote assessment examination and then normalised to a reference score of the past 3 years. This assures that students will not be disadvantaged when compared to previous and future cohorts.

*Freedom of choice*. Because some students may feel uncomfortable with such format or disagree with the normalising procedure, students will be given the freedom to request *a priori,* assessment through oral examination without penalty or prejudice.

*Transparency and emotional support*. Students contributed to the process that resulted in the current assessment model and will be continually consulted throughout delivery. This assures transparency and also significantly decreases emotional distress in a very complex context.

## 3 WHAT LESSONS WERE LEARNED?

We consider that this strategy represents good practice in remote assessment and may be transferable to other contexts and learning environments. So far, the evidence gathered indicates that it helped students gain trust in the assessment process and assures that assessment will achieve its main purpose as a critical tool to improve learning.

